# Patterns of genetic differentiation at MHC class I genes and microsatellites identify conservation units in the giant panda

**DOI:** 10.1186/1471-2148-13-227

**Published:** 2013-10-22

**Authors:** Ying Zhu, Qiu-Hong Wan, Bin Yu, Yun-Fa Ge, Sheng-Guo Fang

**Affiliations:** 1 The Key Laboratory of Conservation Biology for Endangered Wildlife of the Ministry of Education, State Conservation Centre for Gene Resources of Endangered Wildlife, College of Life Sciences, Zhejiang University, No. 388 Yu Hang Tang Road, Hangzhou 310058, PR China

**Keywords:** MHC class I, Genetic differentiation, Conservation, ESUs, MUs, AUs

## Abstract

**Background:**

Evaluating patterns of genetic variation is important to identify conservation units (i.e., evolutionarily significant units [ESUs], management units [MUs], and adaptive units [AUs]) in endangered species. While neutral markers could be used to infer population history, their application in the estimation of adaptive variation is limited. The capacity to adapt to various environments is vital for the long-term survival of endangered species. Hence, analysis of adaptive loci, such as the major histocompatibility complex (MHC) genes, is critical for conservation genetics studies. Here, we investigated 4 classical MHC class I genes (*Aime*-C, *Aime*-F, *Aime*-I, and *Aime*-L) and 8 microsatellites to infer patterns of genetic variation in the giant panda (*Ailuropoda melanoleuca*) and to further define conservation units.

**Results:**

Overall, we identified 24 haplotypes (9 for *Aime*-C, 1 for *Aime*-F, 7 for *Aime*-I, and 7 for *Aime*-L) from 218 individuals obtained from 6 populations of giant panda. We found that the Xiaoxiangling population had the highest genetic variation at microsatellites among the 6 giant panda populations and higher genetic variation at *Aime*-MHC class I genes than other larger populations (Qinling, Qionglai, and Minshan populations). Differentiation index (FST)-based phylogenetic and Bayesian clustering analyses for *Aime*-MHC-I and microsatellite loci both supported that most populations were highly differentiated. The Qinling population was the most genetically differentiated.

**Conclusions:**

The giant panda showed a relatively higher level of genetic diversity at MHC class I genes compared with endangered felids. Using all of the loci, we found that the 6 giant panda populations fell into 2 ESUs: Qinling and non-Qinling populations. We defined 3 MUs based on microsatellites: Qinling, Minshan-Qionglai, and Daxiangling-Xiaoxiangling-Liangshan. We also recommended 3 possible AUs based on MHC loci: Qinling, Minshan-Qionglai, and Daxiangling-Xiaoxiangling-Liangshan. Furthermore, we recommend that a captive breeding program be considered for the Qinling panda population.

## Background

Evolutionary and conservation biologists are concerned with how genetic variation is maintained within populations of endangered species, especially within small and isolated populations [1]. The assumption is that a decrease in genetic variation and a lack of exchange between isolated populations increase the likelihood of extinction by reducing the population's ability to adapt to changing environmental conditions [2].

Generally, biologists use neutral markers (microsatellites) to estimate genetic variation in threatened populations [3,4]. Although variation at neutral markers can provide information about dispersal patterns [5], population connectivity [6], and population history (past demographic expansions or contractions) [2], thus informing decisions regarding the recognition of distinct management units (MUs) [7], these markers cannot provide information on adaptive variation [8]. Such information is necessary in order to designate adaptive units (AUs) for conservation purposes [9]. Hence, adaptive loci should be used in concert with neutral markers to facilitate optimal management decisions [9]. In this study, we consider patterns of variation in major histocompatibility complex (MHC) genes in combination with neutral markers in an effort to understand more about units of conservation associated with the giant panda, *Ailuropoda melanoleuca *[10].

The MHC genes encode molecules involved in immune responses and can be classified into class I and class II genes [11]. Class I genes are mainly associated with intracellular pathogens, such as viruses and protozoa, while class II genes are in charge of extracellular pathogens [12]. MHC class I genes can be further grouped as either classical (class Ia) or nonclassical (class Ib) based on their polymorphisms, expression levels, and functions [13]. Class Ia genes are involved in presenting endogenous peptides to CD8+ cells [14], while class Ib loci have various functions associated with control of natural killer (NK) cell activation [15], successful reproduction [16], and recognition of antigenic lipids [17].

MHC genes (either class I or class II) are highly polymorphic, especially within their antigen-binding region [18]. It is generally believed that balancing selection maintains MHC diversity, which includes overdominant selection and negative frequency-dependent selection [10]. Such variation has been hypothesized to enhance mechanisms of mate choice as well as to provide an adaptive strategy for dealing with new pathogens [19].

**Figure 1:**
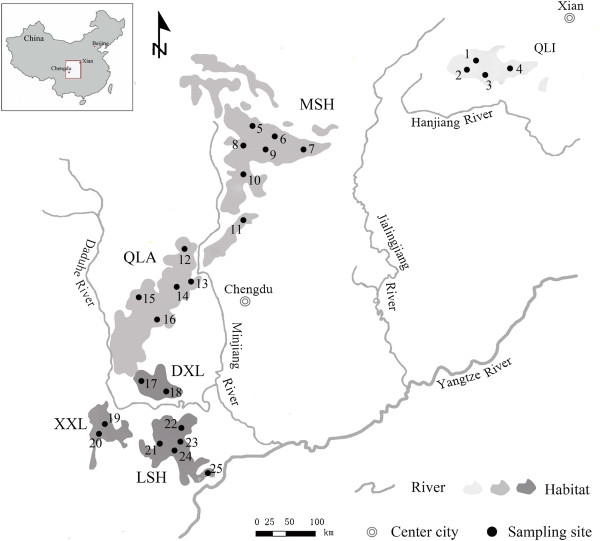
**Habitat distribution and suggested management units of the giant panda.** Habitat distribution of the giant panda, with each filled circle representing a sampling location. Population abbreviations are as follows: QLI, Qinling; MSH, Minshan; QLA, Qionglai; DXL, Daxiangling; XXL, Xiaoxiangling; and LSH, Liangshan. Numbers indicate sampling sites as shown in Additional file 4: Table S3. Different levels of grey in the habitat distribution represent the 3 management units as suggested by this study (light grey, QLI; grey, MSH-QLA; dark grey, DXL-XXL-LSH).

The giant panda (*Ailuropoda melanoleuca*) is a unique endangered species in China. At present, wild populations comprise only about 1500 giant pandas in 6 isolated mountain ranges of China (Figure 1): Qinling (QLI), Minshan (MSH), Qionglai (QLA), Daxiangling (DXL), Xiaoxiangling (XXL), and Liangshan (LSH) [20,21]. These populations are isolated by several rivers (i.e., the Hanjiang, Jianglingjiang, Minjiang, and Dadu rivers; Figure 1) and many roads [21]. The QLI population has been shown to be genetically divergent [22,23], but there is disagreement about whether this population represents a subspecies or a distinct evolutionarily significant unit (ESU) [23]. According to the fossil record, the giant panda originated 3 million years ago (in the early Pleistocene) and was widely distributed from Zhoukoudian in China to northern Burma and northern Vietnam during the middle and late Pleistocene [20]. Seven functional MHC class II genes have been isolated in the giant panda [24,25], and locus-specific genotyping techniques have been established [26,27]. Studies on the MHC class II loci identified moderate levels of allelic diversity and indicated that natural selection and intragenic recombination maintains genetic diversity on MHC class II loci [27]. However, the giant panda appears susceptible to parasites [28,29] as well as several types of viruses associated with domestic animals [30,31]. There is still a need for further investigations of genetic variations at MHC class I genes in this endangered species. Recently, Zhu *et al*. [32] isolated 6 class I MHC genes (i.e., *Aime*-C, *Aime*-F, *Aime*-I, *Aime*-K, *Aime*-L, and *Aime*-1906) from the giant panda, including 4 class Ia genes (*Aime*-C, *Aime*-F, *Aime*-I, and *Aime*-L) and 2 class Ib genes (*Aime*-K and *Aime*-1906), and established locus-specific genotyping techniques for each class Ia gene. Therefore, this pilot study provided an opportunity to examine the adaptive variation of MHC class I genes in structured giant panda populations on a large geographical scale.

In the present study, our aims were to: (1) assess patterns of genetic variation at 4 classical MHC class I genes and 8 microsatellites across 6 extant giant panda populations; and (2) estimate patterns of genetic differentiation among populations and identify conservation units based on both MHC and microsatellite data.

## Results

### MHC variation within and between populations

We obtained 14 exon 2 alleles (4 for *Aime*-C, 1 for *Aime*-F, 5 for *Aime*-I, and 4 for *Aime*-L) and 23 exon 3 alleles (8 for *Aime*-C, 1 for *Aime*-F, 7 for *Aime*-I, and 7 for *Aime*-L) and identified 24 linked long fragment haplotypes (9 for *Aime*-C, 1 for *Aime*-F, 7 for *Aime*-I, and 7 for *Aime*-L) across the 4 *Aime*-MHC class I loci (GenBank: JX987000–JX987023).

The number of haplotypes within the 4 classical *Aime*-MHC class I loci varied among the wild populations, ranging from 17 in QLI to 22 in XXL and LSH (Table 1). Some of these haplotypes were highly abundant in all of the populations (e.g., *Aime*-I*02 and *Aime*-L*02 and 03), while others were detected at very low frequencies and/or only in certain populations (e.g., *Aime*-C*01, 04, and 09; *Aime*-I*05 and 07; and *Aime*-L*05 and 07).

**Table 1:** Haplotypic frequencies, allelic richness, and heterozygosities of MHC class I loci in 6 wild panda populations

		**Population**					
**Locus**	**Haplotype**	**QLI (17)**	**MSH (19)**	**QLA (21)**	**DXL (19)**	**XXL (22)**	**LSH (22)**
*Aime*-C	01	/	0.026	0.016	0.100	/	0.043
	02	0.368	0.250	0.250	0.067	0.107	**0.357**
	03	0.079	**0.355**	**0.375**	0.200	0.196	0.143
	04	/	/	0.031	/	0.018	/
	05	**0.395**	0.066	0.094	0.100	0.107	0.071
	06	0.079	0.105	0.109	**0.467**	0.107	0.157
	07	0.026	0.118	0.063	0.067	**0.375**	0.114
	08	0.053	0.079	0.063	/	0.089	0.071
	09	/	/	/	/	/	0.043
	AR	5.736	6.498	7.018	6.000	6.497	7.519
	*H*_O_	0.684	0.737	0.781	0.933	0.821	0.800
	*H*_E_	0.711	0.785	0.804	0.738	0.792	0.812
*Aime*-F	01	1.000	1.000	1.000	1.000	1.000	1.000
	AR	/	/	/	/	/	/
	*H*_O_	/	/	/	/	/	/
	*H*_E_	/	/	/	/	/	/
*Aime*-I	01	0.125	0.134	0.212	0.250	**0.290**	**0.256**
	02	**0.438**	**0.500**	**0.455**	0.219	0.210	0.207
	03	/	0.073	0.091	0.125	0.129	0.098
	04	0.188	0.232	0.167	**0.375**	0.113	**0.256**
	05	/	0.061	0.030	0.031	0.145	0.073
	06	0.146	/	/	/	0.097	0.073
	07	0.104	/	0.045	/	0.016	0.037
	AR	4.993	4.842	5.528	4.938	6.458	6.613
	*H*_O_	0.626	0.732	0.758	0.625	0.774*	0.829
	*H*_E_	0.741	0.734	0.777	0.756	0.832	0.814
*Aime*-L	01	0.021	0.012	0.121	0.094	0.032	0.049
	02	0.167	0.226	**0.303**	0.219	**0.226**	0.232
	03	0.292	0.262	0.273	0.188	0.210	**0.244**
	04	0.146	**0.286**	0.106	0.156	0.113	**0.244**
	05	/	0.024	0.030	0.031	0.065	/
	06	**0.375**	0.190	0.167	**0.250**	0.129	0.171
	07	/	/	/	0.063	**0.226**	0.061
	AR	4.624	4.946	5.69	6.935	6.663	5.749
	*H*_O_	0.792*	0.810	0.788	0.938	0.774	0.731
	*H*_E_	0.740	0.789	0.803	0.843	0.824	0.802
Total	AR	5.118	5.429	6.079	5.958	6.539	6.627
	*H*_O_	0.701	0.760	0.776	0.832	0.790	0.787
	*H*_E_	0.731	0.769	0.795	0.779	0.816	0.809

Notes: The predominant haplotype of each population is marked in bold. The figures in parentheses are the number of haplotypes in each population. Asterisks denote significant deviations from HWE (*P* < 0.05).

Estimates of heterozygosity revealed higher than expected heterozygosities for DXL and XXL at *Aime*-C, for LSH at *Aime*-I, and for QLI, MSH, and DXL at *Aime*-L. In contrast, other population-locus combinations exhibited lower than expected levels of heterozygosity (Table 1). We only observed significant deviations from Hardy-Weinberg equilibrium (HWE) in the *Aime*-I locus of the smallest population, XXL, and at *Aime*-L in the QLI population; the other combinations all obeyed HWE (Table 1). Different levels of *H*_E_ were found among the wild populations at each locus (*Aime*-C: 0.711–0.812; *Aime*-I: 0.734–0.832; and *Aime*-L: 0.740–0.843). Allelic richness (AR) was also different at 3 polymorphic loci, with *Aime*-C ranging from 5.736 to 7.519, *Aime*-I ranging from 4.842 to 6.613, and *Aime*-L ranging from 4.624 to 6.935 (Table 1). Among the 6 populations across 3 polymorphic MHC loci, the mean *H*_E_ was 0.731–0.816 and the mean AR was 5.118–6.627 (Table 1).

**Figure 2:**
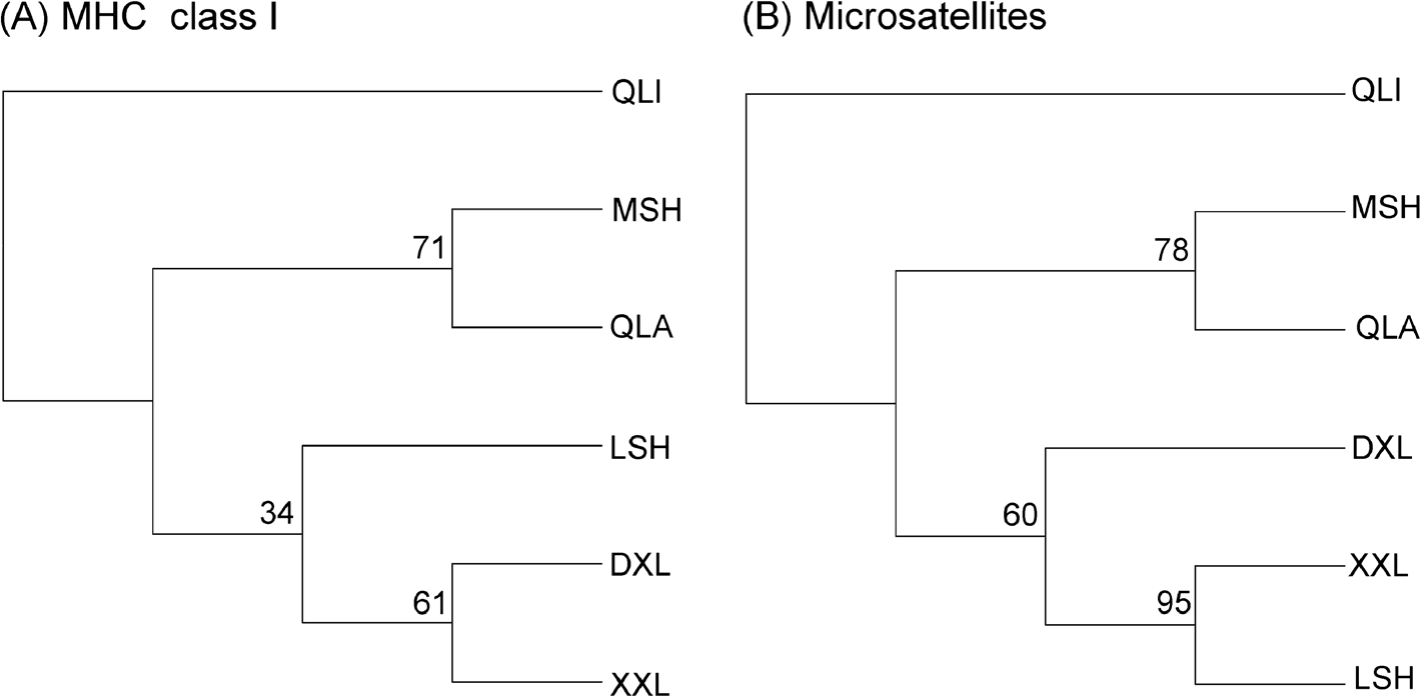
**Neighbor-joining trees.** Neighbor-joining trees of 6 populations based on the F_ST_[51] using MHC class I loci **(A)** and microsatellites **(B)**. The trees are rooted at the midpoint.

All 15 pairwise F_ST_ comparisons revealed there was significant genetic divergence among all populations, with the exception of MSH and QLA (*P* > 0.05; see Additional file 1: Table S1). The neighbor-joining (NJ) tree indicated that the giant panda populations fell into 3 clusters. First, MSH and QLA clustered together with 71% bootstrap values (Figure 2A). Second, The DXL, XXL, and LSH populations clustered together with a weak support of 34% (Figure 2A). Finally, QLI formed the third cluster. F_ST_ values among the 3 clusters are shown in Table 2.

**Table 2:** **F**_**ST **_**index for microsatellites and MHC loci among different groups of giant pandas**

	**QLI**	**MSH-QLA**	**DXL-XXL-LSH**
QLI	/	0.068*	0.066*
MSH-QLA	0.039*	/	0.038*
DXL-XXL-LSH	0.053*	0.015*	/

MHC values are below the diagonal and microsatellites values are above the diagonal. The asterisks indicate *P* < 0.05.

**Figure 3:**
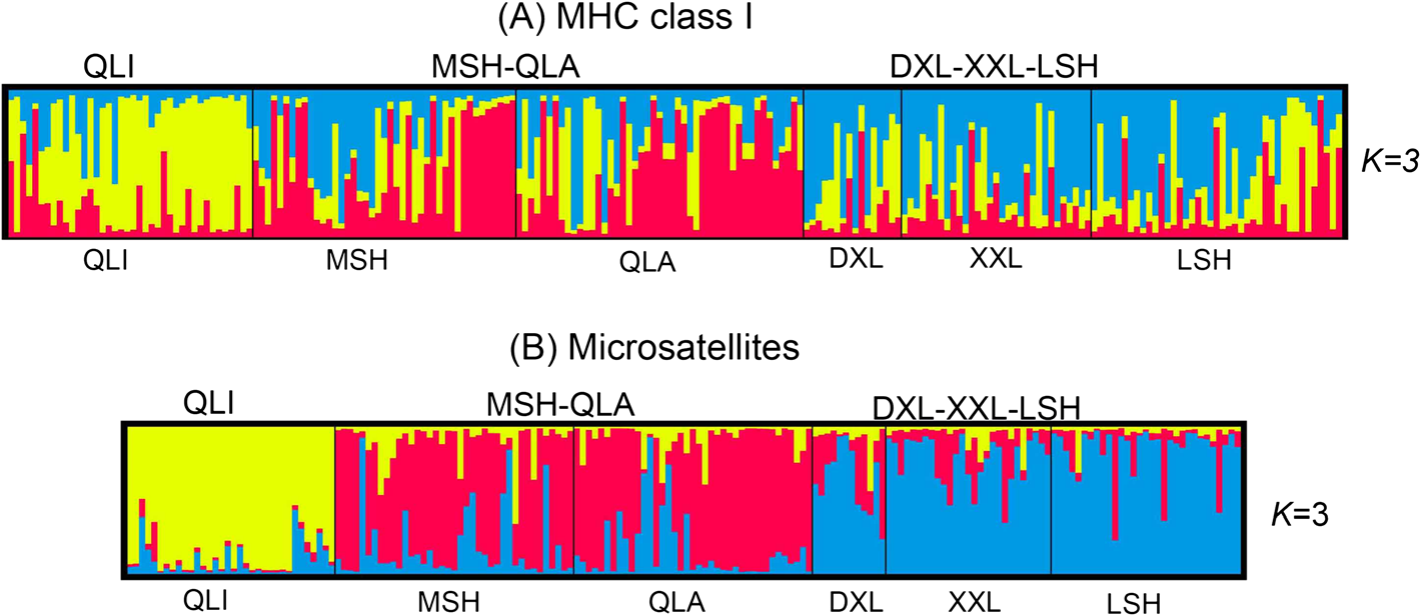
**Bayesian clustering analysis.** Genetic structure of the giant panda based on MHC class I **(A)** and microsatellite loci **(B)** inferred by Bayesian clustering analysis (STRUCTURE) with the sampling location as prior information. Each column represents a single individual. Colors represent different genetic clusters (QLI, yellow; MSH-QLA, red; DXL-XXL-LSH, blue).

Bayesian clustering analysis based on MHC loci also indicate strong subdivision, where the delta k showed 1 peak at K = 3 (see Additional file 2: Figure S1A). QLI (in yellow) was a separate cluster, with the other 2 clusters being MSH-QLA (in red) and DXL-XXL-LSH (in blue; Figure 3). Most of the individuals showed high admixture levels among the 3 clusters.

### Microsatellite variation within and between populations

We identified 121 alleles across 8 microsatellite loci, ranging from 8 to 23 (see Additional file 3: Table S2). Only QLA at *Aime*-3 and GP-4, XXL at *Aime*-10, and LSH at *Aime*-14 significantly deviated from the HWE after Bonferroni correction (see Additional file 3: Table S2). Among the 6 wild populations, XXL showed the highest mean number of alleles (MNA), mean AR, mean *H*_E_, and mean polymorphic information content (PIC) (MNA = 10.8; AR = 8.324; *H*_E_ = 0.856; PIC = 0.832). Effective population sizes (*Ne* values) were estimated for each population, but larger populations (i.e., MSH and QLA) had lower *Ne* values, which were not expected (see Additional file 3: Table S2).

All 15 pairwise F_ST_ comparisons revealed significant genetic differentiation among all pairwise populations, with the exception of DXL and XXL, XXL, and LSH (*P* > 0.05; see Additional file 1: Table S1). The NJ tree showed that the 6 giant panda populations partitioned into 3 clusters. The first cluster contained MSH and QLA (78% bootstrap value), while the second cluster included DXL, XXL, and LSH (60% bootstrap value). QLI formed the third cluster (Figure 2B). F_ST_ values among the 3 clusters are also shown in Table 2.

Bayesian clustering analysis of microsatellite variation indicated the same 3 clusters as MHC (Figure 3 and Additional file 2: Figure S1B). Most of the individuals from the QLI cluster showed very low admixture levels, whereas individuals from the other 2 clusters showed high levels of admixture (Figure 3). The higher admixture levels suggested there was significant gene flow between MSH, QLA, DXL, XXL, and LSH populations. Conversely, low admixture levels demonstrate limited gene flow between QLI and the other populations, indicating that QLI may be suffering from strong genetic isolation. The STRUCTURE plot suggested nearly unidirectional migration from QLI to MSH-QLA (Figure 3), as evidenced by the large proportion of individuals in MSH-QLA that contained substantial QLI heritage (yellow) and the small proportion of individuals in QLI that contained substantial MSH-QLA heritage (red). This movement from QLI, but not into QLI, was in good agreement with previous results [22,33], which showed that the giant panda experienced 2 bottlenecks, the first serious one resulting in a single refuge, QLI, and the second causing 2 refuges, QLI and XXL. The unidirectional movement from QLI to MSH-QLA indicated range expansion followed by the bottlenecks.

Mantel tests revealed that patterns of MHC class I genes and microsatellites were not correlated (r = 0.520, *P* = 0.132), indicating that patterns of MHC class I diversity were not strongly influenced by the effects of stochastic micro-evolutionary processes (migration and drift). Isolation by distance was more obvious for microsatellites than for MHC class I genes (microsatellites: r = 0.703, *P* = 0.022; MHC: r = 0.517, *P* = 0.017).

## Discussion

### Genetic variation levels of *Aime*-MHC class I genes

In this study, we identified 24 exon 2–3 haplotypes for the 4 classical *Aime*-MHC class I genes in 218 wild individuals, averaging 6 haplotypes per locus. In our previous study [32], we detected 13 exon 2 and 16 exon 3 sequences, which formed 17 haplotypes in the Chengdu captive population, revealing that most diversity from wild populations was conserved in captive populations. Compared with the brown bear, the giant panda has similar or fewer MHC class I alleles. A total of 37 alleles (2 pseudo-alleles) were observed from at least 5 loci in 234 brown bear individuals, averaging 7 alleles per locus. However, compared with other endangered felids, *Aime*-MHC class I genes maintain a relatively high level of genetic diversity. For example, a total of 10 alleles (9 functional alleles and 1 pseudo-allele) were detected from 4 putative MHC class I loci in 108 Namibian cheetahs, averaging 2.5 alleles per locus [34]. While 13 putatively functional alleles and one pseudo-allele were found from at least 4 MHC class I loci in 16 highly endangered India Bengal tigers [35]. Furthermore, *Aime*-MHC class II genes also showed higher polymorphism relative to other endangered species [27]. These findings suggested that the giant panda had relative higher genetic variation at their MHC genes, which is necessary for them to cope with changing environmental conditions (e.g., pathogens).

### Genetic variation within populations

According to a survey conducted by the State Forestry Administration of China [21], XXL occupies the smallest habitat area and includes only 32 giant pandas. Interestingly, XXL represented more haplotypes, higher AR, and higher expected heterozygosity at MHC class I genes than those in the larger mountain populations, i.e., MSH, QLA, and QLI (Table 1). Our microsatellite data further revealed that XXL had the highest genetic variation among all of the populations in terms of AR, expected heterozygosity, and number of alleles. Furthermore, a recent MHC II study revealed that XXL has the greatest number of alleles within wild giant panda populations [33]. These results, regardless of adaptive or neutral markers, suggested that the XXL population may have arisen from an ancestral population that had a higher level of genetic diversity, which was also supported by the results of MHC class II study [33]. Although the MSH population covers the largest habitat area and contained 708 individuals as of the last survey round, it did not show the highest level of genetic variation, as was reflected by *Ne* estimates. *Ne* estimates based on microsatellites at 6 populations indicated that MSH had an *Ne* of 90.5, which was smaller than that of the majority of giant panda populations (see Additional file 3: Table S2).

### ESUs, MUs, and AUs in giant panda populations

Population genetics data are useful to identify ESUs, MUs, and AUs in some endangered species [9,36]. In this study, we first defined ESUs in giant pandas in order to protect evolutionarily important groups. Second, we identified MUs in each ESU for management purposes. Finally, we looked for possible AUs to help the government make management decisions. MHC and microsatellite variations in this study revealed that the 6 giant panda populations formed 3 distinct groups. Based on these data, we recommended that the 3 groups be 3 AUs, but partitioned into 2 ESUs, and that one of the ESUs consists of 2 MUs.

The QLI population should be viewed as a separate ESU. Funk *et al.*[9] defined ESU as “a population or group of populations that warrant separate management or priority for conservation because of high genetic and ecological distinctiveness,” and they recommended using neutral and adaptive markers to define ESUs, since neutral and adaptive processes both shape ESUs. Therefore, our recommendation is based on our present genetic data and previous ecological and molecular genetics studies [22,23,37,38]. Our NJ trees based on microsatellite and MHC class I genes revealed that QLI formed a distinct cluster from other populations, which is consistent with our STRUCTURE analysis and previously reported genomic, microsatellite, and DNA fingerprinting data [22,23,37]. The QLI population is currently isolated from other populations by the Hanjiang and Jialingjiang rivers. Additionally, QLI giant pandas live in the south-central range of the QLI Mountain at elevations between 1300 and 2600 m, where the bamboo *Bashania fargesii* (E. G. Camus) Keng f. et Yi grows. In contract, other populations of giant pandas live at elevations of 2100 to 3400 m throughout the year and mainly eat bamboo of the genus Fargesia [20]. Additionally, Wan *et al*. [38] revealed that QLI giant pandas have smaller skulls, larger molars, and different pelage color as compared to other populations’ individuals; these differences may be due to different habitat characteristics in QLI and other mountains. Based on DNA fingerprint and morphological data, Wan *et al*. [22] suggested that the QLI should represent a separate subspecies. However, whether this population represents a subspecies or a distinct ESU is still controversial [23]. Because our evidence indicated that there is significant genetic and ecological distinctiveness between QLI and the other 5 southern populations, we propose that QLI should be a separate ESU and should be monitored and managed separately. Moreover, given that the QLI population has lower genetic diversity at MHC genes and microsatellites and fewer offspring in the captive population compared to the other 5 southern populations, captive breeding of Qinling giant pandas should be encouraged.

The other ESU contains 2 MUs, represented by MSH-QLA and DXL-XXL-LSH. MUs are usually defined as demographically independent populations [36]. If the dispersal rate (m) is smaller than 10%, populations become demographically isolated [39]. Dispersal rate or gene flow is shaped by neutral processes; therefore, neutral markers should be used to define MUs [9]. Our Bayesian clustering analysis using microsatellites showed that 3 clusters existed within giant panda populations. Our results are different from those of a previous study based on microsatellites [23], where they detected 4 clusters (QLI, MSH, QLA, and XXL-LSH). In the present study, MSH and QLA formed 1 cluster, which was confirmed by an NJ tree and was consistent with the data from previously reported DNA fingerprinting and mtDNA analyses [22,40], but was inconsistent with the results of Zhang *et al.*’s study [23]. These inconsistencies could be the result of difference in samples used in the different studies. Three populations, i.e., DXL, XXL, and LSH, formed another cluster, which may not have conflicted with Zhang *et al.*’s study. Because there was only 1 sample collected from the DXL population in the previous study, this sample was considered part of the QLA population for the analysis [23]. The *Ne* values for MSH-QLA and DXL-XXL-LSH were 200 and 300, respectively (see Additional file 3: Table S2). Given that the threshold dispersal rate is 10%, this corresponded to an F_ST_ of ~0.0125 (F_ST_ = 1 / [1 + 4*Ne*m]). The F_ST_ between MSH-QLA and DXL-XXL-LSH was 0.038 (Table 2), which was greater than the threshold of 0.0125; therefore, we can conclude that these 2 clusters should be separate MUs. Moreover, QLI also deserved a separate MU given the greater pairwise F_ST_ between QLI and the other 2 clusters (Table 2). Since MSH and QLA showed no genetic structure among wild populations, we suggest that green corridors should be constructed between these 2 similar populations in order to preserve its existing genetic diversity and evolutionary potential of the populations. In addition, intrapopulation habitat fragmentation is a serious problem for the giant panda [21], so it is essential that we reconnect the patches inhabited by each population in order to enhance contemporary gene flow (individual dispersal) and ensure the long-term survival of the giant panda.

When discussing AUs, adaptive loci should be used [9]. We determined 3 possible AUs (QLI, MSH-QLA, and DXL-XXL-LSH) based on patterns of variation at MHC loci that reflected the ability to adapt to various pathogens. These analyses suggested that QLI should be a separate AU, which was supported by our NJ tree and structure analyses and genomic structure data [37].Our NJ trees revealed that MSH and QLA were most similar (Figure 2A; bootstrap value = 78%); this was supported by our structure analysis and the F_ST_ value between these 2 populations (F_ST_ = 0.003), but was inconsistent with the results of Zhao *et al*. [37]. They detected 3 distinct populations (QLI, MSH, and QLA-DXL-XXL-LSH) based on genomic data. The discrepancy lies in whether MSH and QLA should be together considered as a single AU and could be due to differential sensitivity of these 2 groups of markers. However, given that it is better to use adaptive loci to delineate AUs, it is hard to say whether MSH and QLA should be viewed as separate AUs, though genomic data is much more sensitive than specific genes of known function (i.e., MHC loci) [9]. The genomic structure results reported by Zhao *et al.* were based on all loci [37]. Furthermore, we do not have any data on different types of pathogens within giant panda populations that could directly reflect the different characteristics among possible adaptive groups. Therefore, we can only recommended 3 possible AUs given the above limitations to our data.

## Conclusions

In summary, our work revealed relative high genetic variation at MHC class I genes in the giant panda. Using all loci, we defined 2 ESUs: QLI and MSH-QLA-DXL-XXL-LSH. The differentiation index (FST)-based phylogenetic tree and Bayesian clustering analysis for microsatellite loci suggested the need for 3 MUs: QLI, MSH-QLA, and DXL-XXL-LSH. We recommended 3 possible AUs: QLI, MSH-QLA, and DXL-XXL-LSH based on the patterns of variation in MHC loci. QLI was found to be the most genetically differentiated and had fewer offspring in the captive population, suggesting that captive breeding of pandas from this population should be encouraged. XXL exhibited the highest genetic variation at microsatellites among the 6 giant panda populations and higher genetic variation based on MHC class I genes than that in larger populations (i.e., QLI, MSH, and QLA). Therefore, XXL should be considered before prior to other populations for translocation and captive breeding programs.

## Methods

### Sampling and DNA extraction

We collected 267 samples from 25 geographic locations in 6 segregated mountain ranges (see Additional file 4: Table S3; Figure 1): QLI (n = 40), MSH (n = 43), QLA (n = 47), DXL (n = 25), XXL (n = 51), and LSH (n = 61). These included 35 blood, 109 skin, and 123 faecal samples. Blood samples were obtained from wild-born giant pandas, considered part of the wild population (QLI, MSH, QLA, and LSH). They were collected during routine medical examinations and were stored in liquid nitrogen. Skin samples were obtained from skin tissues from dead wild pandas and were preserved in sealed paper bags in desiccators. The 123 faecal samples (25 DXL, 51 XXL, and 47 LSH; see Additional file 4: Table S3) were collected from nonoverlapping home ranges during the nonreproductive season (between August and November). For faecal samples from the same adjacent home ranges, we performed individual discrimination. First, we performed PCR amplification of 8 microsatellites and 4 *Aime*-MHC-I loci in faecal DNA and found that MHC genes yielded obviously higher amplification success rates than microsatellites. Thus, the faeces were considered to represent a single individual when all alleles were identical across the amplifiable microsatellites and all studied MHC class I loci. Twenty-three faecal samples (18.7%) did not yield PCR products at more than 4 microsatellites and were thus treated as failures of microsatellite-based individualization. We identified individuals from these samples based on genotyping results of 4 *Aime*-MHC-I genes, which nonetheless underwent additional confirmation in an *Aime*-MHC-II-based genotyping analysis conducted in another study [33]. These results allowed us to distinguish 123 faecal samples as having come from 16 giant panda individuals in DXL, 31 in XXL, and 27 in LSH. Thus, we ultimately used 218 individuals for our subsequent analysis (see Additional file 4: Table S3).

Genomic DNA was isolated as described by Wan [26].

### MHC genotyping and haplotyping

We performed locus-specific amplification of the 4 classical *Aime*-MHC class I genes characterized in our previous paper [32]. In addition to separate amplifications of exons 2 and 3, we amplified a long fragment comprising exon 2, intron 2, and exon 3 and used the resulting products to conduct haplotyping (see Additional file 5: Table S4). PCR amplification conditions are presented in Additional file 6: Table S5. A stringent multitube approach was used to obtain reliable genotypes from the faecal samples [41]. If the genotype could not be determined after 2 of 3 amplifications, a fourth was performed. We used single-strand conformation polymorphism and heteroduplex (SSCP-HD) analysis to screen the PCR fragments. Electrophoresis conditions were as described by Zhu *et al*. [32]. In addition to obtaining separate genotypic data from exons 2 and 3, we cloned PCR products representing a longer fragment of exon 2–3 into DH5α competent cells (TaKaRa, Ltd, Dalian, China) and used the recombinants to determine exon 2–3 haplotypes. To identify the combined exon 2–3 genotypes, positive clones were subjected to PCR-SSCP using exon 2- and exon 3-targeted SSCP-series primers. To avoid errors arising from PCR-based recombination, we sequenced at least 8 clones, each showing a unique SSCP banding pattern. If a sequence appeared in at least 2 individuals or was found in 2 independent PCRs from a single individual, we recognized it as an allele.

### Microsatellite genotyping

After assessing their amplification, polymorphism, and yield, we chose 8 giant panda dinucleotide microsatellite loci (see Additional file 7: Table S6) from 37 loci [42-44]. PCR amplification conditions are shown in Additional file 6: Table S5. Genotyping methods were the same as those reported by Li *et al*. [45]. A multitube approach was also used to genotype microsatellite loci, as described above.

### Summary statics

We assessed deviations from HWE and calculated allele frequencies with GenePop 4.0 software [46]. Observed (*H*_O_) and expected (*H*_E_) heterozygosities were obtained from Arlequin 3.1 software [47]. AR, standardized for sample sizes of each locus, was calculated using FSTAT 2.9.3 [48]. Linkage disequilibrium (LD) between pairs of microsatellite loci was evaluated in GenePop 4.0 [46]. We used Micro-Checker to test for the presence of null alleles, stuttering, or large allele dropout for microsatellites [49]. Within the 8 microsatellite markers selected, no evidence was found for LD and/or other genotyping errors for each population. The *Ne* was estimated by the LD method, as implemented in the NeEstimator program [50].

### Estimates of population differentiation

We calculated pairwise F_ST_ values in Arlequin 3.1 [47]. To further assess population structure, we first built NJ trees on the basis of F_ST_ values [51] in PHYLIP 3.69 software [52]. Bootstrap values were obtained by resampling the loci 1000 times. We visualized trees in Figtree 1.4.0 [53], and rooted the trees at the midpoint. We then used Bayesian clustering methods in STRUCTURE V 2.3.3 to detect genetic structure [54]. We conducted 10 runs for K from 1 to 10 with 100,000 burn-in runs from 1,000,000 Markov Chain Monte Carlo (MCMC) operations for each K [54]. Then, the results were uploaded to the online Structure Harvester [55] program, which selects the number of clusters by simultaneously evaluating posterior probability and the delta K statistic of Evanno *et al*. [56]. Graphical output was displayed using DISTRUCT V1.1 [57].

We used Mantel tests to detect whether patterns of population differentiation at MHC and microsatellite loci showed isolation by distance. We first measured geographical distances between different populations by Google Earth [58]. Then, we tested for the relationship between log geographical distance of different populations and G′_ST_/1 – G′_ST_ for 2 markers using a simple Mantel test. The G’_ST_ estimate could control for differences between different markers with different heterozygosities [59]. We conducted Mantel tests in ZT [60].

### Supporting data

The data set supporting the results of this article is available in the Dryad repository [doi:10.5061/dryad.2gt86].

## Competing interests

The authors declare that they have no competing interests.

## Authors’ contributions

SGF conceived the ideas for the study; YFG collected the samples; YZ and BY performed the experiments; YZ and QHW analyzed the data and wrote the manuscript. All authors read and approved the final manuscript.

## Supplementary Material

Additional file 1: Table S1 Population pairwise F_ST_ (A) and G’_ST_ (B) for MHC class I loci and microsatellites. Click here for file

Additional file 2: Figure S1 Mean values of log probability of L(K) and delta K over 10 runs for each K value. (A) MHC class I loci and (B) microsatellites. Click here for file

Additional file 3: Table S2 Levels of genetic diversity estimated from the 8 microsatellite loci for 6 wild populations of giant panda. Click here for file

Additional file 4: Table S3 Information on the giant panda samples analyzed in this study. Click here for file

Additional file 5: Table S4 Locus-specific primers used to amplify exon 2, exon 3, and the longer exon 2–3 fragment from *Aime*-MHC class I genes. Click here for file

Additional file 6: Table S5 Details of PCR conditions for MHC genes and microsatellites. Click here for file

Additional file 7: Table S6 Primers for the 8 polymorphic microsatellite loci used in this study. Click here for file
