# Supplementary material for: Patterns of genetic differentiation at MHC class I genes and microsatellites identify conservation units in the giant panda
Source: BMC Evol Biol. 2013 Oct 22;13:227. doi: 10.1186/1471-2148-13-227 (PMC4015443; doi:10.1186/1471-2148-13-227)
Supplement: Additional file 1: Table S1 — Population pairwise FST (A) and G’ST (B) for MHC class I loci and microsatellites. [file 1471-2148-13-227-S1.doc]

Table S1. Population pairwise F_ST_ (A) and G’_ST_ (B) for MHC class I loci and microsatellites

| A | QLI | MSH | QLA | DXL | XXL | LSH |
| --- | --- | --- | --- | --- | --- | --- |
| QLI | / | 0.064***** | 0.078***** | 0.064***** | 0.061***** | 0.075***** |
| MSH | 0.042***** | / | 0.014***** | 0.035***** | 0.036***** | 0.058***** |
| QLA | 0.038***** | 0.003 | / | 0.039***** | 0.035***** | 0.050***** |
| DXL | 0.074***** | 0.052* | 0.046***** | / | 0.004 | 0.021***** |
| XXL | 0.080***** | 0.055***** | 0.047***** | 0.050***** | / | 0.006 |
| LSH | 0.039***** | 0.023* | 0.023***** | 0.023***** | 0.027***** | / |

| B | QLI | MSH | QLA | DXL | XXL | LSH |
| --- | --- | --- | --- | --- | --- | --- |
| QLI | / | 0.574 | 0.688 | 0.584 | 0.636 | 0.709 |
| MSH | 0.192 | / | 0.198 | 0.344 | 0.271 | 0.490 |
| QLA | 0.234 | -0.011 | / | 0.428 | 0.198 | 0.282 |
| DXL | 0.347 | 0.189 | 0.185 | / | 0.146 | 0.344 |
| XXL | 0.336 | 0.192 | 0.174 | 0.196 | / | 0.115 |
| LSH | 0.170 | 0.091 | 0.109 | 0.083 | 0.091 | / |

MHC values are below the diagonal and microsatellites values are above the diagonal. The asterisks indicate *P* < 0.05 and underlined values denote *P* > 0.05.
