# Supplementary material for: Patterns of genetic differentiation at MHC class I genes and microsatellites identify conservation units in the giant panda
Source: BMC Evol Biol. 2013 Oct 22;13:227. doi: 10.1186/1471-2148-13-227 (PMC4015443; doi:10.1186/1471-2148-13-227)
Supplement: Additional file 2: Figure S1 — Mean values of log probability of L(K) and delta K over 10 runs for each K value. (A) MHC class I loci and (B) microsatellites. [file 1471-2148-13-227-S2.doc]

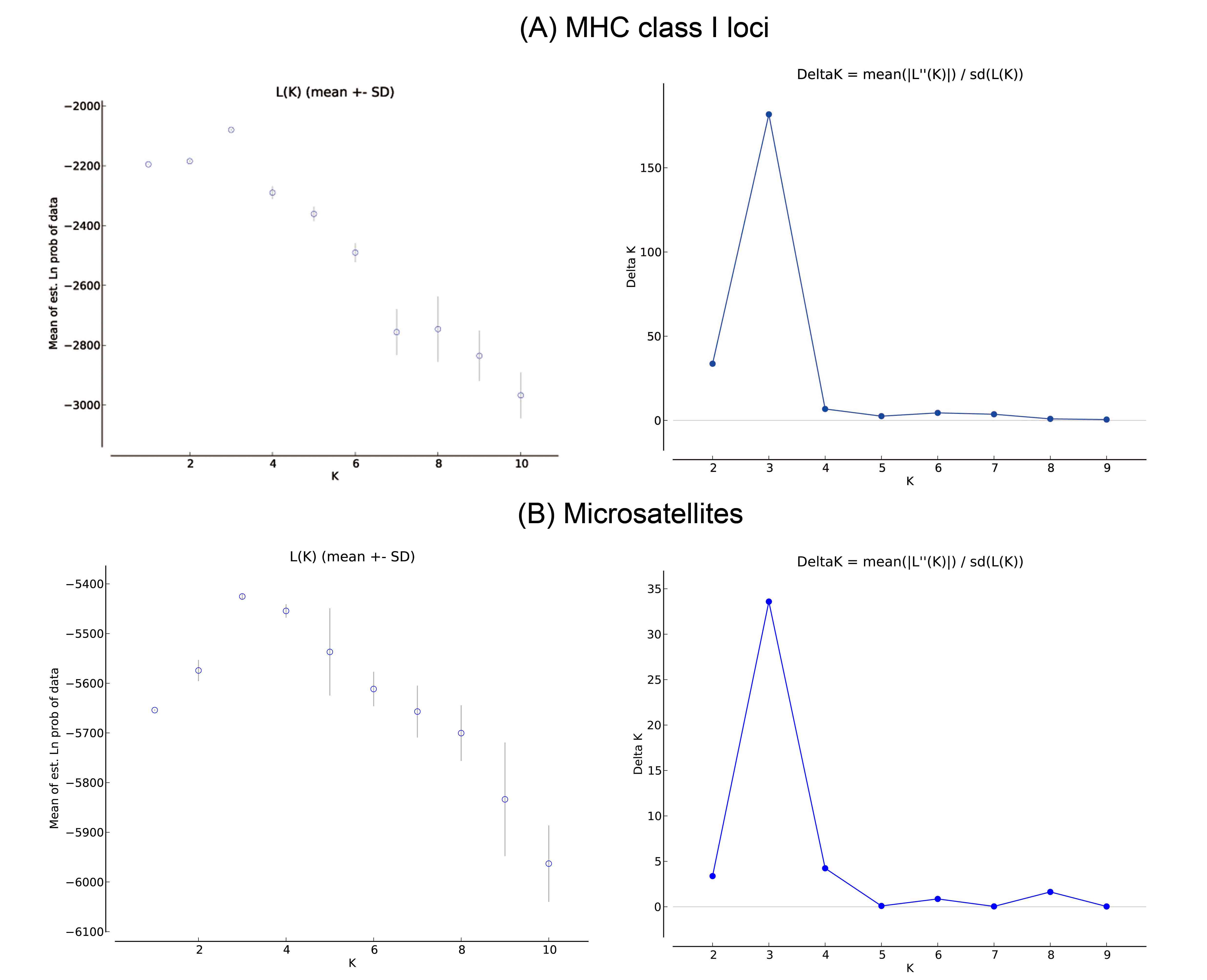


Figure S1. Mean values of log probability of L(K) and delta K over 10 runs for each K value. (A) MHC class I loci and (B) microsatellites.
