# Supplementary material for: Patterns of genetic differentiation at MHC class I genes and microsatellites identify conservation units in the giant panda
Source: BMC Evol Biol. 2013 Oct 22;13:227. doi: 10.1186/1471-2148-13-227 (PMC4015443; doi:10.1186/1471-2148-13-227)
Supplement: Additional file 3: Table S2 — Levels of genetic diversity estimated from the 8 microsatellite loci for 6 wild populations of giant panda. [file 1471-2148-13-227-S3.doc]

Table S2. Levels of genetic diversity estimated from the 8 microsatellite loci for 6 wild populations of giant panda

| Population |  | *Aime*-3  (14) | *Aime*-10  (19) | *Aime*-11  (23) | *Aime*-13  (21) | *Aime*-14  (15) | *Aime*-16  (14) | GP-4  (10) | GP-5  (8) | Mean | *Ne* |
| --- | --- | --- | --- | --- | --- | --- | --- | --- | --- | --- | --- |
| QLI | NA | 6 | 11 | 14 | 9 | 7 | 9 | 5 | 7 | 8.5 | 105 (52-464.2) |
|  | AR | 4.756 | 7.847 | 10.324 | 7.915 | 5.904 | 6.523 | 3.917 | 5.880 | 6.633 |  |
|  | PIC | 0.710 | 0.813 | 0.864 | 0.814 | 0.679 | 0.709 | 0.629 | 0.758 | 0.747 |  |
|  | *H*_O_ | 0.727 | 0.708 | 0.750 | 0.708 | 0.583 | 0.727 | 0.583 | 0.609 | 0.674 |  |
|  | *H*_E_ | 0.728 | 0.852 | 0.883 | 0.851 | 0.729 | 0.712 | 0.707 | 0.789 | 0.781 |  |
| MSH | NA | 9 | 13 | 17 | 16 | 9 | 8 | 6 | 6 | 10.5 | 90.5 (63-152.1) |
|  | AR | 7.507 | 9.605 | 11.219 | 10.009 | 6.663 | 5.479 | 4.294 | 5.106 | 7.5 |  |
|  | PIC | 0.835 | 0.873 | 0.909 | 0.857 | 0.777 | 0.747 | 0.633 | 0.763 | 0.799 |  |
|  | *H*_O_ | 0.756 | 0.780 | 0.821 | 0.763 | 0.683 | 0.684 | 0.558 | 0.744 | 0.724 |  |
|  | *H*_E_ | 0.849 | 0.884 | 0.919 | 0.859 | 0.794 | 0.738 | 0.699 | 0.768 | 0.814 |  |
| QLA | NA | 10 | 9 | 15 | 13 | 6 | 9 | 5 | 5 | 9.0 | 96.1 (56.3-273.1) |
|  | AR | 8.080 | 6.823 | 10.661 | 9.489 | 5.202 | 7.235 | 4.225 | 4.832 | 7.068 |  |
|  | PIC | 0.835 | 0.792 | 0.893 | 0.87 | 0.752 | 0.79 | 0.568 | 0.748 | 0.781 |  |
|  | *H*_O_ | 0.742* | 0.679 | 0.786 | 0.821 | 0.700 | 0.724 | 0.548* | 0.621 | 0.541 |  |
|  | *H*_E_ | 0.865 | 0.796 | 0.906 | 0.881 | 0.785 | 0.801 | 0.647 | 0.768 | 0.806 |  |
| DXL | NA | 9 | 5 | 8 | 10 | 9 | 9 | 4 | 7 | 7.6 | 47.1 (25.3-209.5) |
|  | AR | 8.307 | 4.826 | 8.000 | 9.496 | 8.558 | 7.611 | 3.999 | 6.907 | 7.213 |  |
|  | PIC | 0.834 | 0.688 | 0.8 | 0.883 | 0.854 | 0.748 | 0.722 | 0.838 | 0.796 |  |
|  | *H*_O_ | 0.667 | 0.462 | 0.636 | 0.800 | 0.800 | 0.875 | 0.571 | 0.667 | 0.685 |  |
|  | *H*_E_ | 0.862 | 0.662 | 0.866 | 0.913 | 0.883 | 0.802 | 0.730 | 0.869 | 0.823 |  |
| XXL | NA | 10 | 10 | 17 | 16 | 9 | 9 | 7 | 8 | 10.8 | 103(63.9-238) |
|  | AR | 8.494 | 7.569 | 11.697 | 11.620 | 7.124 | 7.074 | 6.293 | 6.723 | 8.324 |  |
|  | PIC | 0.848 | 0.782 | 0.907 | 0.908 | 0.796 | 0.786 | 0.814 | 0.815 | 0.832 |  |
|  | *H*_O_ | 0.742 | 0.742* | 0.839 | 0.833 | 0.742 | 0.710 | 0.654 | 0.667 | 0.648 |  |
|  | *H*_E_ | 0.876 | 0.819 | 0.928 | 0.925 | 0.833 | 0.821 | 0.819 | 0.829 | 0.856 |  |
| LSH | NA | 11 | 10 | 18 | 12 | 6 | 11 | 7 | 6 | 10.1 | 153.5(92.2-405.1) |
|  | AR | 9.276 | 7.935 | 12.077 | 9.805 | 5.890 | 8.055 | 6.764 | 5.580 | 8.173 |  |
|  | PIC | 0.848 | 0.771 | 0.917 | 0.883 | 0.804 | 0.841 | 0.789 | 0.738 | 0.824 |  |
|  | *H*_O_ | 0.864 | 0.682 | 0.846 | 0.778 | 0.667* | 0.801 | 0.692 | 0.667 | 0.666 |  |
|  | *H*_E_ | 0.890 | 0.798 | 0.922 | 0.901 | 0.829 | 0.855 | 0.857 | 0.768 | 0.853 |  |

The figures in parentheses are the number of alleles for each microsatellite locus. NA: number of alleles; AR: allelic richness; PIC: polymorphic information content; *H*_O_: observed heterozygosity; *H_E_*: expected heterozygosity. Mean: mean value for each project. *Ne:* effective population size for each population. The asterisks indicate *P* values which remained significant after Bonferroni correction for multiple tests with α = 0.05
