# Supplementary material for: Patterns of genetic differentiation at MHC class I genes and microsatellites identify conservation units in the giant panda
Source: BMC Evol Biol. 2013 Oct 22;13:227. doi: 10.1186/1471-2148-13-227 (PMC4015443; doi:10.1186/1471-2148-13-227)
Supplement: Additional file 4: Table S3 — Information on the giant panda samples analyzed in this study. [file 1471-2148-13-227-S4.doc]

Table S3. Information on the giant panda samples analyzed in this study

| Population | No.^a^ | No. of  individuals | Location | Sampling  year | Number and type of samples |
| --- | --- | --- | --- | --- | --- |
| Qinling  (QLI, n = 40) | 1 | 1 | Taibai | 2007 | 1 blood |
|  | 2 | 2 | Yangxian | 1980s, | 2 skin |
|  | 3 | 34 | Foping | 1980s, | 34 skin |
|  | 4 | 3 | Zhouzhi | 1980s, | 3 skin |
| Minshan  (MSH, n = 43) | 5 | 1 | Nanping | 2007 | 1 blood |
|  | 6 | 26 | Wenxian | 1980s, 2007 | 2 blood & 24 skin |
|  | 7 | 2 | Qingchuan | 1980s, 2007 | 1 blood & 1 skin |
|  | 8 | 2 | Songpan | 1980s, | 2 skin |
|  | 9 | 9 | Pingwu | 1980s, 2007 | 4 blood & 5 skin |
|  | 10 | 2 | Beichuan | 2007 | 2 blood |
|  | 11 | 1 | Anxian | 2007 | 1 blood |
| Qionglai  (QLA, n = 47) | 12 | 13 | Wenchuan | 1980s, 2007 | 2 blood & 11 skin |
|  | 13 | 1 | Chongzhou | 2007 | 1 blood |
|  | 14 | 3 | Dayi | 1980s, 2007 | 3 skin |
|  | 15 | 26 | Baoxing | 1980s, 2007 | 16 blood & 10 skin |
|  | 16 | 4 | Lushan | 1980s, 2007 | 1 blood & 3 skin |
| Daxiangling  (DXL, n = 16) | 17 | 15 | Xingjing | 2010 | 22 (15) faeces |
|  | 18 | 1 | Hongya | 2010 | 3 (1) faeces |
| Xiaoxiangling  (XXL, n = 31) | 19 | 15 | Shimian | 2010 | 21 (15) faeces |
|  | 20 | 16 | Mianning | 2010 | 30 (16) faeces |
| Liangshan  (LSH, n = 41) | 21 | 1 | Yuexi | 1980s | 1 skin |
|  | 22 | 9 | Erbian | 1980s, 2007, 2010 | 1 blood & 2 skin 14 (6) faeces |
|  | 23 | 26 | Meigu | 1980s, 2010 | 5 skin & 33 (21) faeces |
|  | 24 | 3 | Mabian | 1980s, 2007 | 1 blood & 2 skin |
|  | 25 | 2 | Leibo | 1980s, 2007 | 1 blood & 1 skin |
| Total |  | 218 |  |  | 267 |

^a^ No. represents numbering of sampling sites and corresponds to the numbers in Figure 1. The figure in parentheses indicates the number of individuals after individualization of faecal samples.
