# Supplementary material for: Patterns of genetic differentiation at MHC class I genes and microsatellites identify conservation units in the giant panda
Source: BMC Evol Biol. 2013 Oct 22;13:227. doi: 10.1186/1471-2148-13-227 (PMC4015443; doi:10.1186/1471-2148-13-227)
Supplement: Additional file 5: Table S4 — Locus-specific primers used to amplify exon 2, exon 3, and the longer exon 2–3 fragment from Aime-MHC class I genes. [file 1471-2148-13-227-S5.doc]

Table S4. Locus-specific primers used to amplify exon 2, exon 3, and the longer exon 2–3 fragment from *Aime*-MHC class I genes. The primer sequences were reported in our previous study .

| Locus | Primer name | Primer sequence (5’-3’) | Size (bp) | Ta (°C) |
| --- | --- | --- | --- | --- |
| *Aime*-CE2 | 1611E21F **^a^** | GCTCTCCCCCACTCAGTA | 600 | 60.1 |
|  | 1611E21R **^a^** | CAGCCGTACATCCACTGGTA |  |  |
| *Aime*-CE3 | 1611E34F **^a^** | ATGACGTATTTCTACACCGGC | 1127 | 64.1 |
|  | 1611E33R **^a^** | TGCAGGTCTAAGAGGGAGAGCGCT |  |  |
| *Aime*- C | 1611E21F | GCTCTCCCCCACTCAGTA | 1255 | 60.0 |
|  | 1611E33R | TGCAGGTCTAAGAGGGAGAGCGCT |  |  |
| *Aime*-FE2 | 152E2A3 **^a^** | GCCCTGCTCTCCCCCACTCAG | 496 | 65.3 |
|  | 152E2A4 **^a^** | CGGGGGTTCCTGAGAGTTGGGGC |  |  |
| *Aime*-FE3 | 152E3A1 **^a^** | AGTCCGAGCGTTGCCCCAACTCTC | 787 | 59.5 |
|  | 152E3A2 **^a^** | AGTAATGGCCCTGAGTAAGGTTTC |  |  |
| *Aime*-F | 152E2A3 | / | 1247 | 60.0 |
|  | 152E3A2 | / |  |  |
| *Aime*-IE2 | 128E22F **^a^** | CCTGCTCTCCCCAACGCG | 614 | 62.4 |
|  | 128E22R **^a^** | GTCACAGCCGTGCATCCA |  |  |
| *Aime*-IE3 | 128E31F **^a^** | CCGAGTGGACTTGCAGACCGCC | 1131 | 62.5 |
|  | 128E32R **^a^** | GTCCGGGGTTTCTGAAGAAGAACG |  |  |
| *Aime*-I | 128E22F | / | 1467 | 60.0 |
|  | 128E32R | / |  |  |
| *Aime*-LE2 | 1300E22F **^a^** | GGGAGAAGGGTCGGGCGGGAC | 535 | 62.2 |
|  | 1300E21R **^a^** | AGGTCACAGCCGTGCATCTC |  |  |
| *Aime*-LE3 | 1300E31F **^a^** | GCTCTCACACCATCCAGGA | 887 | 62.1 |
|  | 1300E31R **^a^** | GTCGGGGGTTTCTGAAGAAGAATC |  |  |
| *Aime*-L | 1300E22F | / | 1354 | 60.0 |
|  | 1300E31R | / |  |  |
| *Aime*-C/F/I/LE2 | C1E2B1 **^b^** | TCAGCCCCTCCGCGCCCGCAG | 312 | 66.3 |
|  | C1E2B2 **^b^** | GACCCGGGCCGCGTCGCTCAC |  |  |
| *Aime*-C/F/I/LE3 | C1E3B1 **^b^** | TCGCCTCCTGTCGGGCGGGGCCAG | 349 | 65.6 |
|  | C1E3B2 **^b^** | AGCCAGCCCCAGCGGAGGGG |  |  |

^a^ and ^b^ indicate the first and second primer pairs used for the nested PCR, respectively. The 4 classical loci used the same second-round primer pairs.

1. Zhu Y, Sun DD, Ge YF, Yu B, Chen YY, Wan QH: **Isolation and characterization of class I MHC genes in the giant panda (*Ailuropoda melanoleuca*)**. *Chinese Sci Bull* 2012, **57**:1-8.
