# Supplementary material for: Patterns of genetic differentiation at MHC class I genes and microsatellites identify conservation units in the giant panda
Source: BMC Evol Biol. 2013 Oct 22;13:227. doi: 10.1186/1471-2148-13-227 (PMC4015443; doi:10.1186/1471-2148-13-227)
Supplement: Additional file 6: Table S5 — Details of PCR conditions for MHC genes and microsatellites. [file 1471-2148-13-227-S6.doc]

Table S5. Details of PCR conditions for MHC genes and microsatellites

| Marker | Reaction component |  | PCR condition |
| --- | --- | --- | --- |
| MHC | 2×GC Buffer (Mg^2+^) | 5μl | 5 min at 95°C followed by 22-25 cycles of 30s at 95°C, 30s at the annealing temperature (Table S4), and 1 min at 72°C, with a final extension period of 5 min at 72°C |
| The first-round PCR reaction | dNTPs (2.5 mM) | 0.2 mM |  |
|  | Forward / Reverse primer (10μM) | 0.2 μM |  |
|  | Ex-Taq (5U/μl) | 0.25 U |  |
|  | BSA (when templates are skin and fecal) | 0.05μl |  |
|  | gDNA | 20 ng |  |
|  | ddH_2_O | To 10μl |  |
| MHC | 10×Ex-Taq Buffer (Mg^2+^) | 3μl | denaturation at 95°C for 5min, followed by 30-35 cycles of 30s at 95°C, 30s at the annealing temperature, and 30s at 72°C, and ended with an elongation step at 72°C for 5 min |
| The second-round PCR reaction | dNTPs (2.5 mM) | 0.6 mM |  |
|  | Forward / Reverse primer (10μM) | 0.6μM |  |
|  | Ex-Taq (5U/μl) | 0.3μl |  |
|  | Template (products of the first-round PCR) | 1μl |  |
|  | ddH_2_O | To 30μl |  |
| Microsatellite | 10×Ex-Taq Buffer (Mg^2+^) | 1μl | denaturation at 95°C for 5min, 35-45cycles of 95°C for 30s at optimized annealing temperature for 35s, 72°C for 45s, a final extension at 72°C for 10min. |
|  | dNTPs (2.5 mM) | 0.2 mM |  |
|  | Forward / Reverse primer (10μM) | 0.2 μM |  |
|  | IRD labeled M13 primer (LI-COR). | 2μM |  |
|  | Ex-Taq (5U/μl) | 0.25 U |  |
|  | BSA (when templates are skin and fecal) | 0.05 |  |
|  | gDNA | 20 ng |  |
|  | ddH_2_O | To 10μl |  |
