# Supplementary material for: Patterns of genetic differentiation at MHC class I genes and microsatellites identify conservation units in the giant panda
Source: BMC Evol Biol. 2013 Oct 22;13:227. doi: 10.1186/1471-2148-13-227 (PMC4015443; doi:10.1186/1471-2148-13-227)
Supplement: Additional file 7: Table S6 — Primers for the 8 polymorphic microsatellite loci used in this study. [file 1471-2148-13-227-S7.doc]

Table S6. Primers for the 8 polymorphic microsatellite loci used in this study

| Locus | Repeat motif | Primer sequences (5′–3′) | References |
| --- | --- | --- | --- |
| *Aime*-3 | (AC)_23_ | F:TCCCCAGTTAGCT TCTCG  R:AGGCTTGCTTTATGTCGG |  |
| *Aime*-10 | (AG)_10_ | F: ATACCTGGAAACTTCATA  R: GGTATTTTGTCCTTAGCG |  |
| *Aime*-11 | (TTTC)_11_(TC)_21_ | F:ATGAAAGAGTGCAAGTAAA  R:CTGGCAAAAGGTGAATGT |  |
| *Aime*-13 | (AC)_10_(AG)_23_ | F:TTAGCCTCCTGGGTATT  R:ACAAGTGCGTGGGAAGTA |  |
| *Aime*-14 | (TC)_23_ | F:TCTGACCCTCCCCTACTA  R:TCTCCTTTGATTTTCTATC |  |
| *Aime*-16 | (AG)_30_ | F:TTTACGCTTGGCACTCTA  R:TTGGGAAATCATGGCTCT |  |
| GP-4 | (CA)12 | F:CCTGGCATAATGTGAGCAAC  R:AGGATGTGGAGACCACGACT |  |
| GP-5 | (GT)16 | F: TCAGACCCTAGATTTCATTC  R: GAAGAGCCATACCACAGAG |  |

1. Zhang HM, Guo Y, Li DS, Wang PY, Fang SG: **Sixteen novel microsatellite loci developed for the giant panda (*Ailuropoda melanoleuca*)**. *Conserv Genet* 2009, **10**(3):589-592.

2. Wu H, Zhan XJ, Zhang ZJ, Zhu LF, Yan L, Li M, Wei FW: **Thirty-three microsatellite loci for noninvasive genetic studies of the giant panda (*Ailuropoda melanoleuca*)**. *Conserv Genet* 2009, **10**(3):649-652.
